# AGING STEREOTYPES MODERATE THE RELATIONSHIP BETWEEN COSTS AND DISCOUNTING

**DOI:** 10.1093/geroni/igz038.2992

**Published:** 2019-11-08

**Authors:** Claire Growney, Erica L O’Brien, Jesse DeLaRosa, Thomas M Hess

**Affiliations:** North Carolina State University, Raleigh, North Carolina, United States

## Abstract

Aging is associated with normative declines in cognitive resources that increase the costs associated with mobilizing resources in cognitively demanding activities. Selective Engagement Theory (Hess, 2014) hypothesizes that changes in costs influence the motivation to engage in such activities in everyday life. We used an economic discounting task to examine the relationship between both objective estimates (systolic blood pressure responses) and subjective estimates (NASA Task Load Index) of cognitive costs in 78 older adults’ (ages 64-85) decisions to engage in more or less demanding activities. Perceptions of costs were meaningfully tied to actual costs (SBP), but further influenced by personal or primed attitudes about aging. Interestingly, decisions to engage in demanding activities—as reflected in discounting decisions—were less influenced by effort expenditure in the activity than by perceptions of difficulty. These results underscore the role that negative stereotypes play in undermining motivation to engage in potentially beneficial activities.

